# Edible Green Solvent for Optimized Catechins Extraction from Green Tea Leaves: Anti-Hypercholesterolemia

**DOI:** 10.26502/fjppr.053

**Published:** 2022-06-29

**Authors:** Kazutoshi Fujioka, Taher A. Salaheldin, Kavitha Godugu, Harold V. Meyers, Shaker A. Mousa

**Affiliations:** 1 The Pharmaceutical Research Institute, Albany College of Pharmacy and Health Sciences, 1 Discovery Drive, Rensselaer, NY 12144 USA.; 2 Shifa Biomedical Corporation, Malvern, PA 19355 USA

**Keywords:** green tea, catechins, EGCG, extraction, chitosan, edible solvent, dyslipidemia, hypercholesterolemia

## Abstract

Catechin polyphenols are the major bioactive ingredients in green tea with various human health benefits. Extraction of catechins from green tea (GTE) leaves at optimized standard conditions is still a challenging approach. An optimized, rapid, and economic extraction method is industrially needed. We hypothesized that certain extraction techniques in the presence of natural polymers and antioxidants might improve GTE catechin extraction yield and its biological activity. The effect of microwave (30–60 seconds irradiation in a typical kitchen microwave) assisted extraction (MAE) and ultrasonic assisted extraction (UAE) techniques were evaluated separately and in combination. To study the effect of the extraction solvent, nine edible green solvent combinations were investigated namely water, ascorbic acid, chitosan/ascorbic acid, carboxymethylcellulose /ascorbic acid, methylcellulose /ascorbic acid, chitosan/methylcellulose/ascorbic acid, methylcellulose, chitosan/acetic acid, and ethanol. The amounts of extracted catechins from green tea leaves were quantified with HPLC-UV.

Data showed that the use of MAE & UAE technique was the optimal in producing a higher extraction yield of catechins. Chitosan/ascorbic acid was the optimized solvent with high extraction efficiencies of catechins. Studies in high fat diet fed animals demonstrated significant reduction of total cholesterol and LDL-C by GTE after 3 weeks of oral daily administration. In conclusion, efficient extraction, and stabilization of catechins from green tea leaves demonstrated a significant lowering of high fat diet-mediated elevation in blood cholesterol and LDL-C levels.

## 1. Introduction

Green tea, one of the most widely consumed beverages in the world, is an aqueous infusion of dried leaves of “Camellia Sinensis”. The birthplace of green tea is in Asia. Although abundant foliage is produced by the tea plant, only the two leaves and the buds at each young shoot are picked for tea [1, 2]. Green tea consists of polyphenols, such as flavonoids, (e.g., catechins, and tannins), free amino acids, alkaloids, (e.g., caffeine), ascorbic acid, saponins, and unsaturated fatty acids. Catechins constitute a major component of green tea (30% of the chemical composition), which include epicatechin (EC), epigallocatechin (EGC), epicatechin gallate (ECG), and epigallocatechin gallate (EGCG) [3]. Epigallocatechin gallate (EGCG) is the major catechin of green tea (constitutes 9–13% weight of green tea) and exhibits multiple health benefits due to its antioxidant nature [4]. Catechins have a wide range of beneficial health effects, such as reducing the risk of cancer, anti-inflammatory, reducing the risk of developing diabetes mellitus, infertility, anti-hypercholesterolemia, antiatherosclerosis, antiulcer, ability to inhibit human platelet aggregation, reducing skin wrinkling, and in reducing the risk of cardiovascular diseases [5,6].

**Figure 1:**
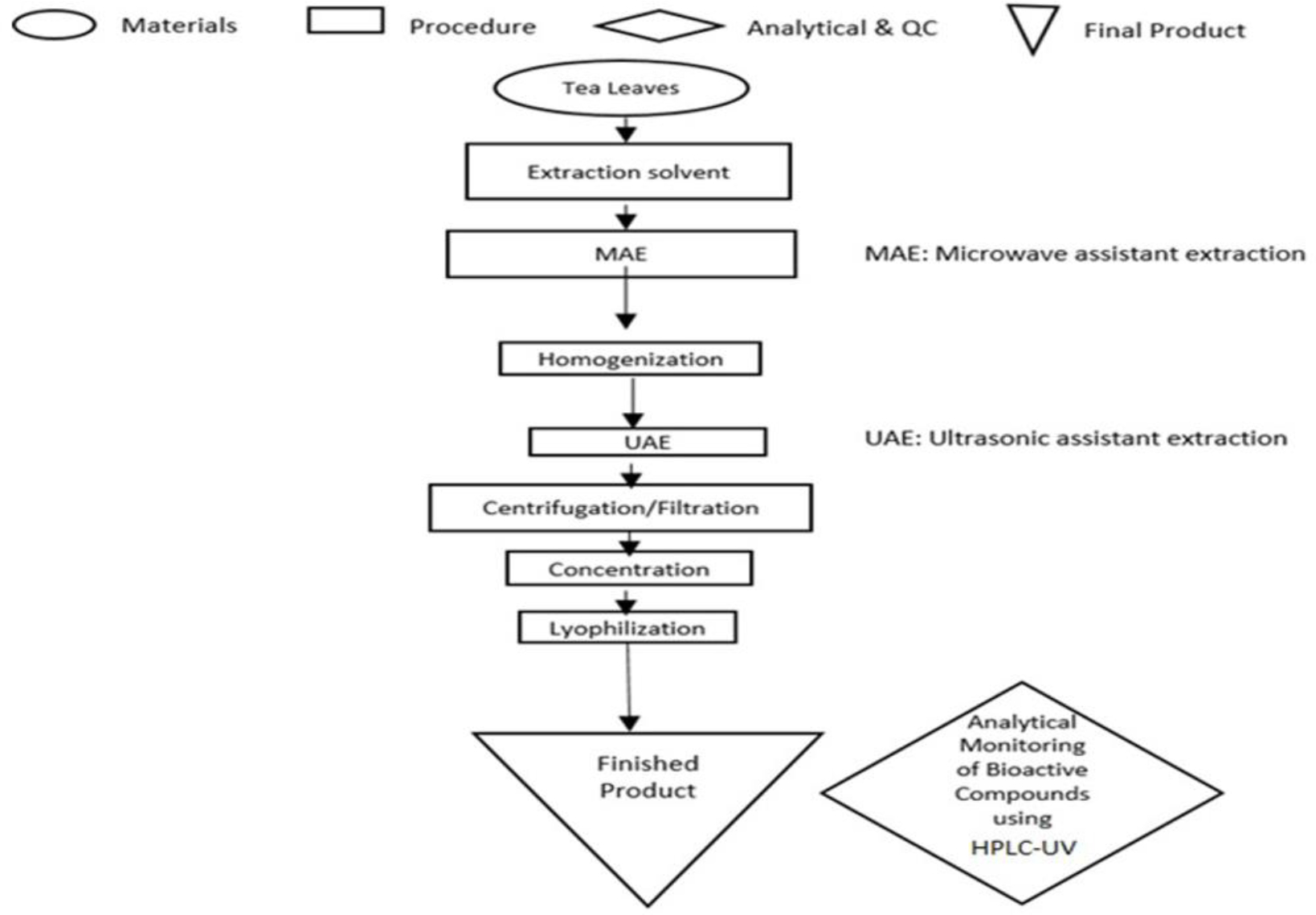
Schematic diagram for extraction protocol for catechins from green tea leaves.

Different parameters affect the extraction efficiency of the active ingredients from green tea, their chemical nature, storage time and conditions, and the presence of interfering substances [7]. The solubility of the flavonoids depends on the polarity of the solvent used, degree of polymerization, interaction with other food constituents, and the formation of insoluble complexes [8–10]. There is no standardized or completely satisfactory procedure that is suitable for the extraction of all flavonoids or specific types of flavonoids in green tea [11,12]. The most common extraction protocols are conventional solvent extraction combined with mechanical or thermal techniques, such as ultrasound assisted extraction (UAE) [13,14], microwave assisted extraction (MAE) [15,16], high hydrostatic pressure (HHPE) [17], and supercritical fluid extraction (SFE) [18]. Polar solvents, (e.g., methanol, ethyl acetate, acetone, and water), are common conventional solvents for extraction; and their combinations are also used. The extraction period usually varies from 1 min to 24 hrs. Reportedly, high temperature (more than 100 °C) and prolonged extraction time (more than 2 hours) lead to the degradation of catechins due to partial epimerization of epigallocatechin gallate (EGCG) and epicatechin gallate (ECG) into gallocatechin gallate (GCG) and catechin gallate (CG), respectively. Therefore, it is necessary to extract catechins from green tea leaves at as low temperature as possible. Ultrasonic assisted extraction (UAE) is suggested for improved sensory attributes because it is carried out at a low temperature avoiding volatile component evaporation and thermal degradation of active biomolecules. Meanwhile, microwave assisted extraction (MAE, at 60–80 °C for 5 min) is suggested for improved extractability of polyphenols with reduced time and energy consumption. It is well known that catechins are unstable polyphenolic compounds; and therefore, their protection from oxidation during the extraction process is strongly needed. The present work aims to optimize an extraction protocol for phytochemicals in green tea leaves by selecting the best extraction technique (UAE and/or MAE) and solvent composition, which leads to a high extraction yield in a short time (Figure 1). We introduce novel edible green extraction solvents that exhibit the required polarity and preservative storage network of natural polymers, including chitosan, ascorbic acid, carboxymethylcellulose (CMC), methylcellulose (MC), and their combination (Table 1).

We hypothesized that the extracted catechins in the presence of ascorbic acid and chitosan will be simultaneously adsorbed inside the cavities of the polymer network leading to preservation of its stability and biological activities. In addition, the multivalent functional groups of the polymer offer

## 2. Materials and Methods

### 2.1 Chemicals

Green tea leaves (Organic certified, Gunpowder loose leaves, Davidson’s, Reno, NV, USA), (+)-catechin (C), (−)-epicatechin (EC), (−)-epigallocatechin (EGC), (−)-gallocatechin gallate (GCG), (−)-epicatechin gallate (ECG), (−)-epigallocatechin gallate (EGCG), gallic acid, caffeine, theobromine, theophylline, L-ascorbic acid, chitosan (50,000 – 150,000 Da), carboxymethylcellulose (CMC), methylcellulose (MC), ethanol, acetic acid and Amicon ultra-0.5 centrifugal filters, were purchased form Sigma-Aldrich (St. Louis, MO, USA).

### 2.2 Extraction procedure

Phytochemicals are extracted with nine extraction solvents (Table 1) using extraction techniques, i.e., UAE, MAE, and MAE/ UAE.

### 2.3 Effect of extraction technique

#### 2.3.1 Ultrasonic assisted extraction (UAE)

Five grams of green tea leaves were dispersed in 200 mL of deionized water and left to brew under stirring at 37 °C for 2 hours. Then the mixture was homogenized at 20,000 rpm homogenization speed (PT3100D, Polytron, Kinematica, Bohemia, NY, USA) for 10 min until a homogenous viscous dark different types of electrostatic attraction sites for the unstable active ingredients and provide a stable protective environment [19]. We then evaluated hypo-cholesterolemic effects of orally administered GTE obtained by the optimized method on blood total cholesterol and LDL-C in high fat diet fed mice. green mixture was obtained. After homogenization it was sonicated using a ultrasonication probe at 80 μm amplitude (Q55, Qsonica LLC, Newtown, CT, USA) for 10 min. The mixture was double filtered using 45 μm and 22 μm membrane filters; and a clear golden solution was obtained. The solution was transferred into freeze drying vessels, frozen at −80 °C overnight, and lyophilized (MD85, Millerick technology, Kingston, NY, USA) to obtain green tea extract powder [13,14].

#### 2.3.2 Microwave assisted extraction (MAE)

Five grams of green tea leaves were dispersed in 200 mL of deionized water and proceeded for microwave digestion at 60–80 °C for 5 min. The mixture was homogenized at 20,000 rpm homogenization speed (PT3100D, Polytron, Kinematica) for 5 min until a homogenous viscous dark green mixture was obtained. The mixture was double filtered using 45 μm and 22 μm membrane filters and a clear golden solution was obtained. The solution was transferred into freeze drying vessels, frozen at −80 °C overnight, and lyophilized (MD85, Millerock technology,) to obtain green tea extract powder [16].

#### 2.3.3 Combined MAE and UAE (MAE/ UAE)

Five grams of green tea leaves were dispersed in 200 mL of deionized water and proceeded for microwave digestion at 60–80 °C for 5 min. The mixture was homogenized at 20,000 rpm homogenization speed (PT3100D, Polytron) for 5 min until a homogenous viscous dark green mixture was obtained. After homogenization the mixture was sonicated using a ultrasonication probe at 80 μm amplitude (Q55, Qsonica) for 10 min. The mixture was double filtered using 45 μm and 22 μm membrane filters and a clear golden solution was obtained. The solution was transferred into freeze drying vessels, frozen at −80 °C overnight, and lyophilized (MD85, Millrock technology) to obtain green tea extract powder. All of the extracted powders were weighed and compositions of catechins in the extracts were quantitatively analyzed with high performance liquid chromatography-UV spectrometry (HPLC-UV).

#### 2.3.4 Effect of extraction solvent

For high extraction throughput, we used the combined microwave assisted extraction (MAE, 60–80 °C for 5 min) and ultrasound assisted extraction (UAE) technique (MAE/ UAE). Nine extraction solvents were tested, namely water, ascorbic acid, chitosan/ascorbic acid, carboxymethylcellulose (CMC)/ ascorbic acid, methylcellulose (MC)/ascorbic acid, chitosan/methylcellulose (MC)/ascorbic acid, methylcellulose (MC), chitosan/ acetic acid, and 50% ethanol. Table 1 summarizes the compositions of extraction solvents. Green tea leaves (5 grams) were added to 200 mL of extraction solvent and underwent microwave digestion at 60–80 °C for 5 min. The mixture was homogenized at 20,000 rpm homogenization speed (PT3100D, Polytron) for 5 min until a homogenous viscous dark green mixture was obtained. After homogenization the mixture was sonicated using ultrasonication probe at 80 μm amplitude (Q55, Qsonica) for 10 min. The mixture was double filtered using 45 μm and 22 μm membrane filters and a clear golden solution was obtained. The solution was transferred into freeze drying vessels, frozen at −80 °C overnight, and lyophilized (MD85, Millrock technology) to obtain green tea extract powder. Figure 1 schematically summarizes the extraction protocol. Quantitative analytical monitoring of bioactive compounds was conducted using HPLC-UV (Waters, Milford, MA, USA). The best extraction protocol (high catechins concentration) was chosen for nano formulation preparation, which will be used for the further in vitro and in vivo biological evaluations (the results of biological evaluations will be published elsewhere).

### 2.4 HPLC sample preparation

Standard calibration solutions of EGCG, EGC, ECG, epicatechin, catechin, GCG, caffeine, theobromine, theophylline, and gallic acid solutions were prepared in 50% MeOH in water at concentrations of 500, 400, 300, 200, 100, 10, and 1 ug/mL. Two grams of green tea extracts (GTEs) were dissolved in 1 mL of 50% MeOH in water. After vortex-mixing for 30 min, mixtures were centrifuged for 15 min; and aliquots of supernatant (100 uL) were used for HPLC-UV analysis in duplicate.

### 2.5 Quantitative analysis of catechins by HPLC-UV

**Figure 2:**
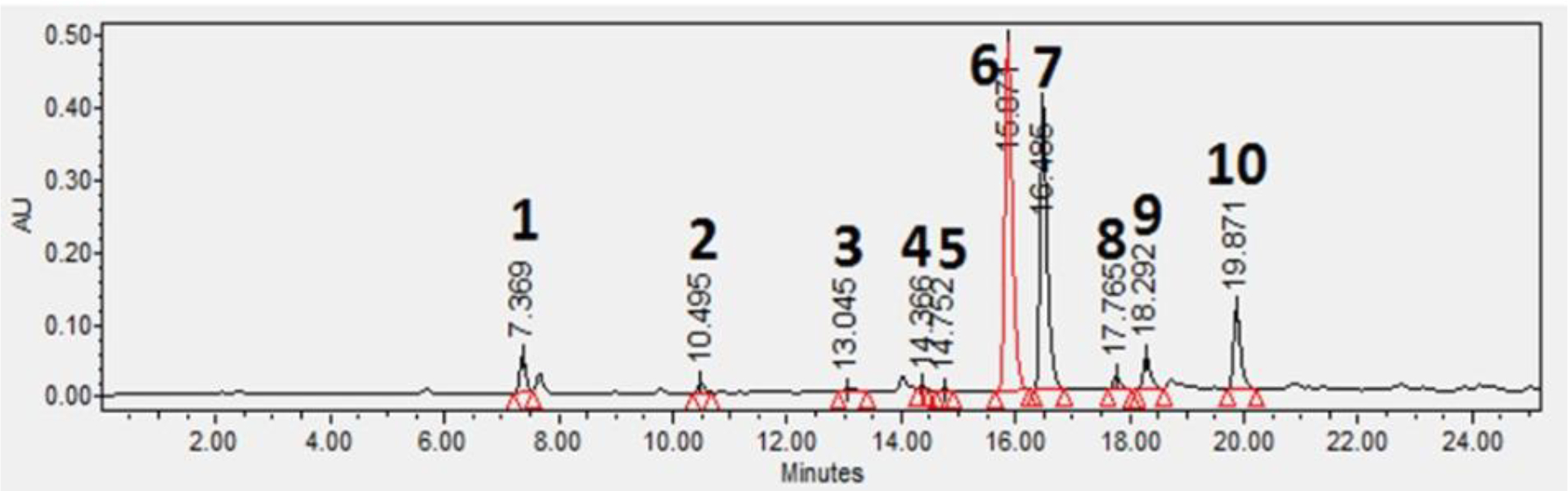
HPLC-UV chromatogram of phytochemicals in green tea extract, 1: Garlic acid, 2: Theobromine, 3: Theophylline, 4: EGC, 5: Catechin, 6: Caffeine, 7: EGCG, 8: GCG, 9: EC, 10: ECG

A Waters 2695 Separations Module (Waters) equipped with a Waters 2996 Photo Diode Array Detector was used for HPLC analysis. A Pursuit XRs 3 C18 column (150 × 4.6 mm, Agilent, Santa Clara, CA, USA) was used for separation in the reversed-phase mode. The software used for operating the HPLC and analyzing data was Empower 3 Software (Waters). Mobile phases were water containing 0.1 % formic acid (A) and methanol (B). The flow rate was 1.0 mL/min. The gradient was linear from 5% B at 0 min to 95% B at 40 min and held for 5min. The column temperature was at room temperature; and the injection volume was 10 μL. UV spectra were obtained from 210–400 nm and used for identification of analytes. Quantification of phytochemicals was carried out using peak areas at a wavelength of 275 nm of UV chromatograms with an external standard method [20]. A typical UV chromatogram is shown in Figure 2. Accuracy was estimated with analysis of standard solutions at concentrations of 500, 100, and 10 μg/mL (n=3). The limit of quantification (LOQ) was determined using data of six injections of a standard solution (10 μg/mL). LOQ was calculated using the standard deviations (SD) of signals for the analyte and the slope of linear regression curve as follows:


LOQ=10×SD/slope

The HPLC-UV method for standard solutions was linear (r > 0.99) from a concentration of 10 to 500 μg/mL for all analytes. The accuracy for the analytes was more than 90% and less than 110%. The LOQ was equal to or less than 10 μg/mL for all analytes under the current conditions.

### 2.6 Animals

The in vivo studies were conducted at the animal facility of the Veterans Affairs Medical Center (VAMC), Albany, New York according to the guidelines of NIH and the institutional guidelines for humane animal treatment. The applied animal protocols were approved by the Institutional Animal Care & Use Committee (IACUC) at VAMC.

### 2.7 Pharmacodynamic study using nutritionally induced hypercholesterolemia model

High fat diet male C57BL/6 mice aged 4–5 weeks and weighing 20–25 g were purchased from Taconic Biosciences, Inc. (Germantown, NY, USA). Animals were maintained under specific pathogen-free conditions and housed under controlled conditions of temperature (20–24°C) and humidity (60–70%) and 12 h light/dark cycle with ad libitum access to water and high fat diet. Mice were fed a high-fat irradiated diet (TD.**D12492**, Research Diets, Inc., New Brunswick, NJ, USA) that provides 60% kcal from fat sources to increase total cholesterol. The composition of the diet is shown in Table 2.

The mice were grouped into two arms (n=3) and administrated orally daily with water for vehicle control and GTE, (30 mg/kg) for 3 weeks. Plasma was collected after week 3 treatment to monitor the level of LDL-C as measured enzymatically. Blood samples (100 μl) were collected from the retro-orbital venous plexus via heparinized capillary tubes containing 2 USP units of ammonium heparin per tube (Carolina, Burlington, North Carolina, USA). Plasma was separated immediately using centrifugation (5,000 × g) for 5 min at room temperature and then kept at −80°C until assayed for lipid profile. Plasma total cholesterol and LDL-C levels were measured with the cholesterol and LDL assay kit according to the manufacturer’s instructions (Abcam, Boston, MA, USA).

### 2.8 Statistical analysis

Statistical analysis was performed using GraphPad Prism (San Diego, CA, USA). All data are presented as mean ± standard error of the mean. The t-test t-was used to determine differences among the groups. ***P <0.001, **P <0.01, *P <0.05 were considered as significant.

## 3. Result

### 3.1 Effect of extraction technique

The extraction technique is one of the most important factors that could affect the yield of biomolecules extracted from green tea leaves. Table 3 represents the results of quantitative HPLC-UV analysis of extracted bioactive ingredients using three extraction techniques, i.e., UAE, MAE, and combined MAE/UAE, which were described in the methodology section. The results of quantification showed that extraction techniques have drastic impacts on the yield of catechins from green tea leaves. The yield of phytochemicals increased with the combined extraction technique (MAE/ UAE). For example, (−)-epigallocatechin gallate (EGCG), which is the dominant catechin, 80–90%, increased by four-fold compared to UAE technique and by two-fold compared to MAE technique. From the viewpoint of stability, ultrasonic assisted extraction (UAE) is the preferred method for catechins extraction due to the extraction process conducted at lower temperatures that avoid the degradation that would occur at high temperatures. The main disadvantage of UAE is that it needs prolonged extraction time to accomplish high yields, which explain the low concentrations of the extracted active ingredients with UAE in this study.

### 3.2 Effect of extraction solvent

The extraction solvent is another key element affecting the extraction yield of catechins in addition to the extraction technique. We, therefore, investigated the effect of nine solvents on the extraction efficiency of catechins by the combined MAE/ UAE technique (Table 3).

### 3.3 Pharmacodynamic study using nutritionally induced hypercholesterolemia model:

**Figure 3:**
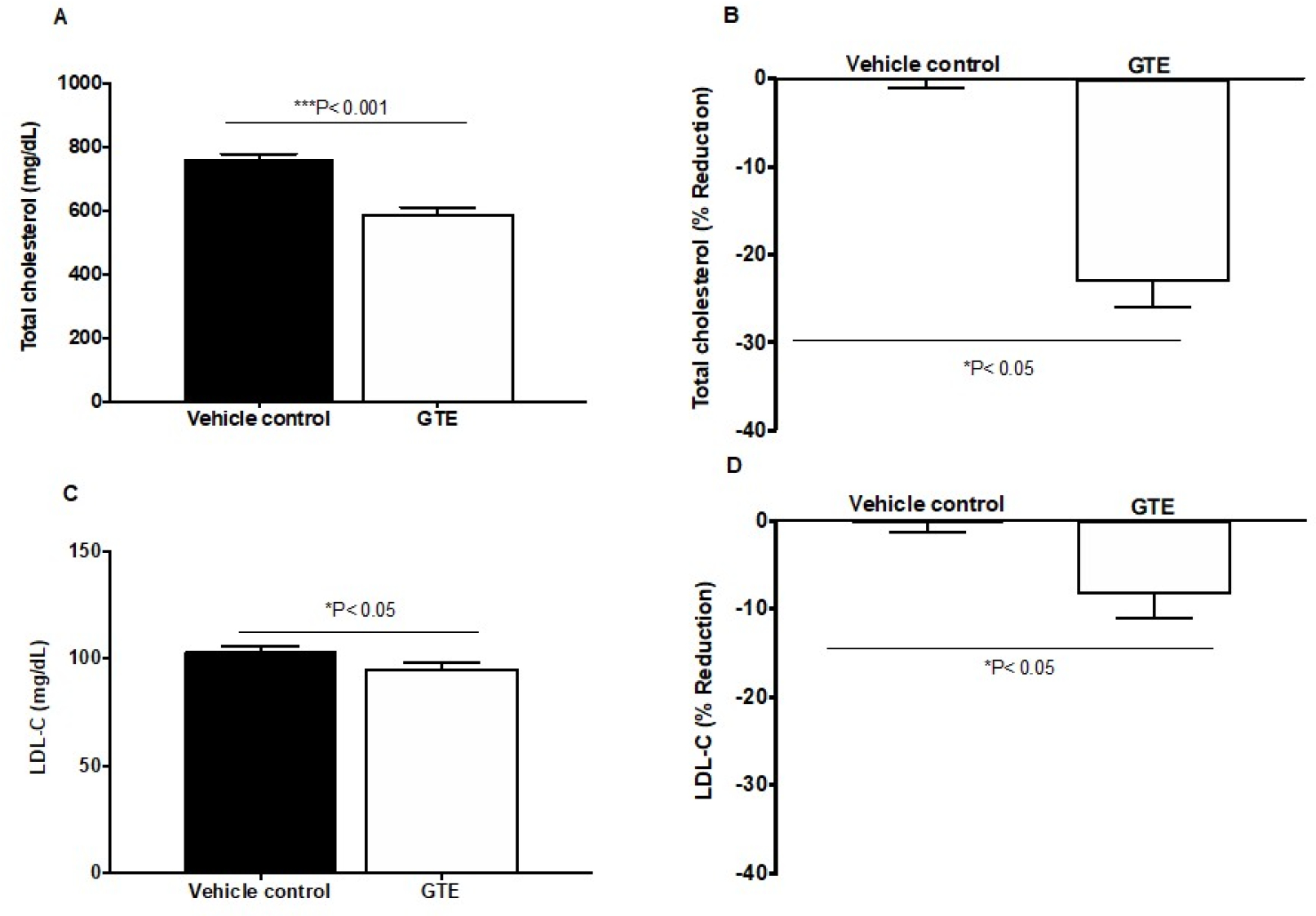
Levels of total cholesterol and LDL-C in plasma from a high-fat diet mice fed for 3 weeks and treated with A) GTE administered daily orally with water at 30 mg/kg versus vehicle control given water orally for 3 weeks. Blood was collected at week 3. B) Percentage reduction of plasma total cholesterol. C) LDL-C levels in vehicle control versus GTE treated daily for 3 weeks. D) Percentage reduction of plasma LDL-C was compared with control. Values are presented as mean ± S.D. Statistical analysis conducted using t-test to determine differences among the groups. GTE was statistically significant compared to vehicle control.

Animals were fed the high-fat diet and randomly assigned to one of the different groups such that the average of each biomarker level is comparable among the different groups. Plasma total cholesterol, free cholesterol [Note: no free cholesterol data shown in Fig. 3] and LDL-C levels were measured. For plasma total cholesterol, mice treated with green tea extract (GTE) showed 25% total cholesterol reduction compared to vehicle group after three weeks oral treatment, Figure 3 A and B. Plasma LDL cholesterol (LDL-C) showed significant (*P<0.0.5) reduction in GTE was 15% compared to the control group after three-weeks treatment, as illustrated in Figure 3, C&D.

## 4. Discussions

Our hypothesis was validated and accepted where indeed certain extraction techniques in the presence of natural polymers and antioxidants improved GTE catechin extraction yield, stability, and its biological activity. In MAE, the microwave oven generates electromagnetic radiation, which is absorbed by the water molecules in the extraction medium and by the green tea biomass, produces a drastic rise in temperature that disrupts the cell wall of the green tea leaves, and increases the solubility and diffusion coefficient of the bioactive ingredients [21–23]. The results in this study showed that the high temperature affected the yield of the extracted bioactive ingredients. The yield of catechins, alkaloids and gallic acid increased two-fold, however with MAE as compared to UAE, and this finding agrees with previous studies [23]. The main limitation of MAE is the high temperature, which can cause a drastic degradation of tea bioactive ingredients [24]. Therefore, it is necessary to optimize the extraction at a temperature as low as possible. The combination of MAE (60–80 °C) and UAE (MAE/ UAE) has dual benefits of the lower temperatures of ultrasonic waves by UAE and microwave heating by MAE, which maximize the extraction yield and minimizes the extraction time as shown in Table 3. The combined MAE/ UAE technique proved to be the best extraction technique method to maximize the efficiency of the extraction solvent. This combined protocol will impel us to overcome the limitations of high temperature, time consuming methods and low extraction efficacy.

The results show that the chitosan/ascorbic acid protocol is the best extraction solvent composition for catechins from green tea leaves compared to the other solvents. It is interesting to note that this solvent composition had a strong impact on the yield of individual catechins and the yield of each catechin was affected differently by the extraction solvent compositions (Table 4). Similar observations were observed with caffeine, theobromine, theophylline, and gallic acid. It is known that the charge and the polarity of solvents influence the efficiency of the catechins extraction process. Catechins are electronegative polar molecules and attracted to the electropositive polar chitosan molecules; and such positive polarity of the chitosan network enhances the molecular interactions with catechins [25]. Ascorbic acid has a bifunctional role on extraction efficiency. It increases the polarity of the extraction medium due to the presence of four hydroxyl groups that will facilitate the dissolution of catechins in addition to its antioxidant activity that protects catechins from further oxidation and increases their stability [26].

Epidemiological studies have shown an inverse association between coronary heart disease (CHD) risk and green tea consumption in humans [27]. Such lipid lowering action of green tea was attributed to its catechins content which inhibits the intestinal absorption of ingested lipids [28], and it inhibits PCSK-9 activity and up-regulates the LDL-R in liver tissues [29]. Hence, preservation of the polyphenol functional groups in GTE bioactive catechins protects the hypo-cholesterolemic effects of GTE bioactive polyphenols.

## 5. Conclusion

We optimized the extraction technique and the solvent composition for phytochemicals in green tea leaves. The combined technique of microwave assisted extraction and ultrasonic assisted extraction (MAE/ UAE) was the optimized technique producing higher extraction yield compared to each technique separately. Also, the edible green solvent, chitosan/ascorbic acid in water was the optimized solvent with the highest extraction efficiency of catechins among the nine solvents invistigated, which can be attributed to the high adsorbing capability of the chitosan network and the anti-oxidative protection of ascorbic acid for extracted catechins. Therefore, the combined MAE/ UAE technique with a chitosan/ascorbic acid solvents system is a rapid, scalable, and optimized approach for efficient extraction of catechins from green tea leaves that, preserves the hypo-cholesterolemic effects of GTE bioactive polyphenols.

## Figures and Tables

**Table 1:** Extraction solvent compositions

	Extraction Protocol	Solvent composition
1	Water (control)	Deionized water
2	Ascorbic acid	1% Ascorbic acid in water
3	Chitosan/ascorbic acid	1% Ascorbic acid and 0.5% Chitosan in water
4	Carboxymethylcellulose (CMC)/ ascorbic acid	1% Ascorbic acid and 0.5% CMC in water
5	Methylcellulose (MC)/ascorbic acid	1% Ascorbic acid and 0.5% MC in water
6	Chitosan/Methylcellulose/ascorbic acid	1% Ascorbic acid, 0.25% MC and 0.25% chitosan in water
7	Methylcellulose (MC)	0.5% MC in water
8	Chitosan/acetic acid	1% Acetic acid and 0.5% chitosan in water
9	Alcohol extract	50% Ethanol in water

**Table 2:** Diet formula

**Product #**		**D12492**	

		**gm%**	**kcal%**
Protein Carbohydrate Fat		26.2	20
	**Total** **kcal/gm**	26.3	20
		34.9	60
		5.24	100
		
**Ingredient** Casein, 80		**gm**	**kcal**
		
Mesh L-Cystine			
		200	800
Corn Starch Maltodextrin		3	12
10 Sucrose		0	0
Cellulose, BW200		125	500
Soybean Oil Lard*		68.8	275.2
Mineral Mix, S10026 DiCalcium		50	0
Phosphate Calcium Carbonate		25	225
Potassium Citrate, 1 H2O		245	2205
Vitamin Mix, V10001 Choline		10	0
Bitartrate		13	0
FD&C Blue Dye #1		5.5	0
		16.5	0
		10.0	40
		2	0
		0.05	0

**Total**		**773.85**	**4057**

Note: Table 2 needs to be fixed to align all of the ingredients with g% and kcal%. Also, i) there are only 12 listed ingredients but 15 rows of values for g%/kcal%. ii) For ‘Protein Carbohydrate Fat’, what are the 4 rows of values? There’s no labels for each set of values. iii) ‘Product #’ header – there are no listed product no.’s, so this header should be changed

**Table 3:** HPLC results of green tea extracted bioactive compounds (mg/g green tea extract) by UAE, MAE, and combined MAE and UAE techniques

	UAE	MAE	MAE/ UAE
**EGCG**	34.84 ± 0.03	77.71 ± 0.07	142.80 ± 0.13
**EGC**	5.90 ± 0.03	7.94 ± 0.04	10.54 ± 0.06
**ECG**	2.22 ± 0.14	4.12 ± 0.27	7.39 ± 0.48
**Epicatechin**	0.32 ± 0.01	0.67 ± 0.01	0.78 ± 0.01
**Catechin**	0.16 ± 0.01	0.54 ± 0.05	0.74 ± 0.06
**GCG**	0.25 ± 0.02	0.70 ± 0.02	1.43 ± 0.02
**Caffeine**	6.35 ± 0.02	7.12 ± 0.03	8.81 ± 0.03
**Theobromine**	0.036 ± 0.002	0.096 ± 0.002	0.108 ± 0.002
**Theophylline**	0.064 ± 0.001	0.096 ± 0.005	0.378 ± 0.33
**Gallic acid**	0.198 ± 0.009	0.336 ± 0.019	0.636 ± 0.038

**Table 4:** HPLC analysis of bioactive compounds in green tea extracts (mg/g green tea extract) Values are presented as mean value ± standard deviation (SD)

	Water	Ascorbic acid	Chitosan/ascorbic acid	CMC / ascorbic acid	MC / ascorbic acid	Chitosan / MC/ascorbic acid	Chitosan / MC	Chitosan / acetic acid	50 % Alcohol
EGCG	142.8±0.1	99.636±0.09	232.24±0.21	13.248±0.01	25.6434 ± 0.02	44.8842 ± 0.04	8.96±0.01	65.2776 ± 0.06	174.8278 ± 0.16
EGC	10.536 ± 0.09	17.25±0.09	39.36±0.21	1.152±0.00	15.3102 ± 0.08	14.7224 ± 0.08	1.456±0.01	0.3304±0.00	0.6342±0.00
ECG	7.392±0.29	4.508±0.29	14.8±0.95	0.576±0.04	1.2324±0.08	2.0554±0.13	0.336±0.02	4.2952±0.28	11.8384±0.76
Epicatechin	0.672±0.01	0.874±0.01	5.2±0.07	0.576±0.01	0.1422±0.002	0.239±0.003	0.112±0.002	1.7464±0.02	1.7516±0.02
Catechin	0.744±0.07	0.368±0.03	1.04±0.09	0.192±0.01	0.3792±0.03	0.3346±0.03	0.728±0.06	0.8024±0.7	0.6644±0.06
GCG	0.696±0.02	3.174±0.02	9.52±0.02	0.288±0.02	2.5122±0.02	2.3422±0.02	0.112±0.02	0.0944±0.02	0.2416±0.02
Caffeine	8.808±0.03	12.512±0.05	47.44±0.19	21.504±0.08	11.5656 ± 0.04	11.0896 ± 0.04	13.72±0.05	14.7736 ± 0.06	17.3046±0.07
Theobromine	0.096±0.002	0.092±0.002	0.72±0.001	0.096±0.002	0.0948±0.002	0.0956±0.002	0.728±0.001	0.1416±0.001	0.3926±0.0004
Theophylline	0.096±0.005	0.828±0.077	0.4±0.035	0.768±0.071	0.5688±0.052	0	0.504±0.045	0.0944±0.004	0.2114±0.16
Gallic acid	0.336±0.019	0.782±0.048	4.24±0.28	3.84±0.25	0.7584±0.046	0.7648±0.047	1.064±0.067	1.0384±0.065	1.2986±0.082

Note: adjust Table 4 columns and row sizes to ensure all letters of the chemical names or on the same line

## Data Availability

All raw data are available at the Pharmaceutical Research Institute upon request.

## List of abbreviations:

C: Catechin
CG: Catechin gallate
CMC: Carboxymethylcellulose
EC: Epicatechin
ECG: Epicatechin gallate
EGC: Epigallocatechin
EGCG: Epigallocatechin gallate
GCG: Gallocatechin gallate
HHPE: High hydrostatic pressure
HPLC-UV: High performance liquid chromatography-UV spectrometry
MAE: Microwave assisted extraction
MC: Methylcellulose
SFE: Supercritical fluid extraction
UAE: Ultrasonic assisted extraction
LDL-C: Low density lipoprotein-cholesterol
